# Sit-to-Stand (STS) Movement Analysis of the Center of Gravity for Human–Robot Interaction

**DOI:** 10.3389/fnbot.2022.863722

**Published:** 2022-05-30

**Authors:** Haiyan Wang, Shanshan Xu, Jiayu Fu, Xiangrong Xu, Zhixiong Wang, Ri Su Na

**Affiliations:** ^1^Osaka Institute of Medicine and Engineering, Maanshan University, Ma'anshan, China; ^2^Department of Medicine, Graduate School of Osaka University, Suita, Japan; ^3^School of Mechanical Engineering, Anhui University of Technology, Ma'anshan, China

**Keywords:** STS, auxiliary stand robot, center of gravity, optical gait acquisition system, AMTI

## Abstract

As the aging process is springing up around the whole world, the problem of sit-to-stand (STS) for the elderly has become the focal point of older people themselves, their families, and society. The key challenge in solving this problem is developing and applying the technology of auxiliary robots for standing up and auxiliary stands. The research is not only to find the appropriate fundamentals from a healthcare perspective in the center of gravity movement (COM) curve of the elderly during STS but also to meet the psychological needs of the elderly during STS in a comfortable, pleasant, and safe way. To obtain the skeleton tracking technology used in this study, we used the Vicon optical motion capture system and automatic moving target indicator (AMTI) 3-D force measuring table to obtain a COM curve of the elderly during STS. The stationary process, speed, and sitting posture analysis during STS were combined in this paper to seek a solution to the psychological needs of the elderly. The analysis is conducted with the integration of medicine, engineering, art, and science into this research. Finally, a movement curve and related dynamic parameters that can truly reflect the STS of the elderly are obtained, and the discussion is provided. The research result can provide theoretical and technical support for the later development of auxiliary robots for standing up and auxiliary stands technology products, so that the elderly can STS comfortably, happily, and safely with the assistance of human–robot interaction.

## Introduction

In the recent years, the problem of the aging population has intensified, and some social issues caused by aging are also increasing (China Pension Network, 2018), especially among the disabled elderly (Zhu, 2016; Wang et al., 2018). The demand for elderly care and medical services is under increasing pressure. Services such as life care and spiritual comfort are in urgent need of development, which brings new problems and opportunities for human health management and services and has attracted the attention of researchers in many disciplines such as management, medicine, mechanics, and intelligent engineering. In particular, management and care services for sit-to-stand (STS) movements are vital in daily life, which is one of the most important factors that contribute to severe injuries in the activities of the elderly. Due to muscle weakness, knee injuries, spinal cord injuries, and lower limb venous thrombosis, it is sometimes difficult for older adults to complete simple STS movements. So, regular STS movements cause specific injuries to the elderly physically and mentally (Kamnik et al., 2005; King, 2010). Therefore, from the perspective of health management and services, it is crucial to standardize standing movement and design auxiliary standing equipment and facilities and leisure nursing tube requirements (Qiu-hui et al., 2018).

From the perspective of managing and serving human health, we hope to study, design, test standing trajectories and explore their safety according to the requirements of the elderly. Consequently, we can provide a basis for the future design of sets of sport-assisted standing movement equipment to help the elderly stand and design equipment control systems and demonstrate mechanical structure functions (Lo et al., 2010; Min and Shuo, 2013; Rafii, 2014; Yuxuan, 2016). Such research is based on integrating emotion and engineering to improve the comfort, confidence, and pleasure of standing for the elderly by studying the sit-to-stand process. Simultaneously, this research is also based on the integration of medical specialties to assist the elderly in standing through engineering means from the perspective of health management and services to solve the difficulties in standing for the elderly (Hernandez, 2015).

According to research, sit-to-stand movement is the foundation of daily life, such as getting up, going to the bathroom, and sitting up. It requires people to change from a sitting to a standing posture. The average person performs STS movements 60 ± 22 times a day. Normal older people in the Chinese community have more STS movements than those in hospitals and rehabilitation wards (Dall and Kerr, 2010). Due to the deterioration of muscle strength, joint range of motion, and balance in the elderly, they cannot perform certain movements like normal people, thus increasing the risk of STS movements in daily life. We need to creatively address the STS issues for older adults and patients in a new research perspective to meet the needs of a large population. In particular, we need to help older adults build confidence from the integration of emotion and movement while addressing their living issues to achieve a comfortable and safe STS environment.

In addition, data show that the increasing aging in China is also putting tremendous pressure on society to provide care. Approximately 79.1% of older adults suffer from more than one chronic disease. These individuals generally require nursing care, in some cases long-term care, placing great demands on families, society, and the government. Only 37.5% of men over the age of 80 can cook for themselves (Bai, 2010). In the future, assistive robotics or assistive technology is the key to solving such problems (Xiaoyu and Kaixuan, 2010), especially the STS problem.

University of Delaware, USA; Australian Institute of Re-Rehabilitation; University of Ljubljana, Slovenia; Chine port, an Italian company; Osaka University School of Medicine and Tokyo Electric and Communication University, Japan; Harbin Institute of Technology, Zhejiang University, and the Hefei University of Technology, China, etc., have invested a lot of time and effort and made great efforts (Kamnik et al., 2005; Heqing, 2011; Lihong, 2016; Zhou, 2016; Wang et al., 2018). However, there is still a long way to cahieve a safe, comfortable, and pleasant STS process, which is far from the abovementioned health management and service requirements. Without taking into account the safety and comfort of human movements, the bulky operating system that is not easy to handle, and the large size that is not easy to move, the existing deficiency is the failure to find an auxiliary robot and auxiliary technology that can be set up to consider the movement curves of the above factors during STS. The basis for solving this problem is to find the trajectory of the center of gravity during STS, which can both complete the STS process smoothly and ensure the safety of the STS process. At the same time, it has good comfort for the elderly, so that the elderly who have difficulty in STS can sit and stand confidently and happily, and the STS process can meet the requirements of health management and service level.

**Figure 1:**
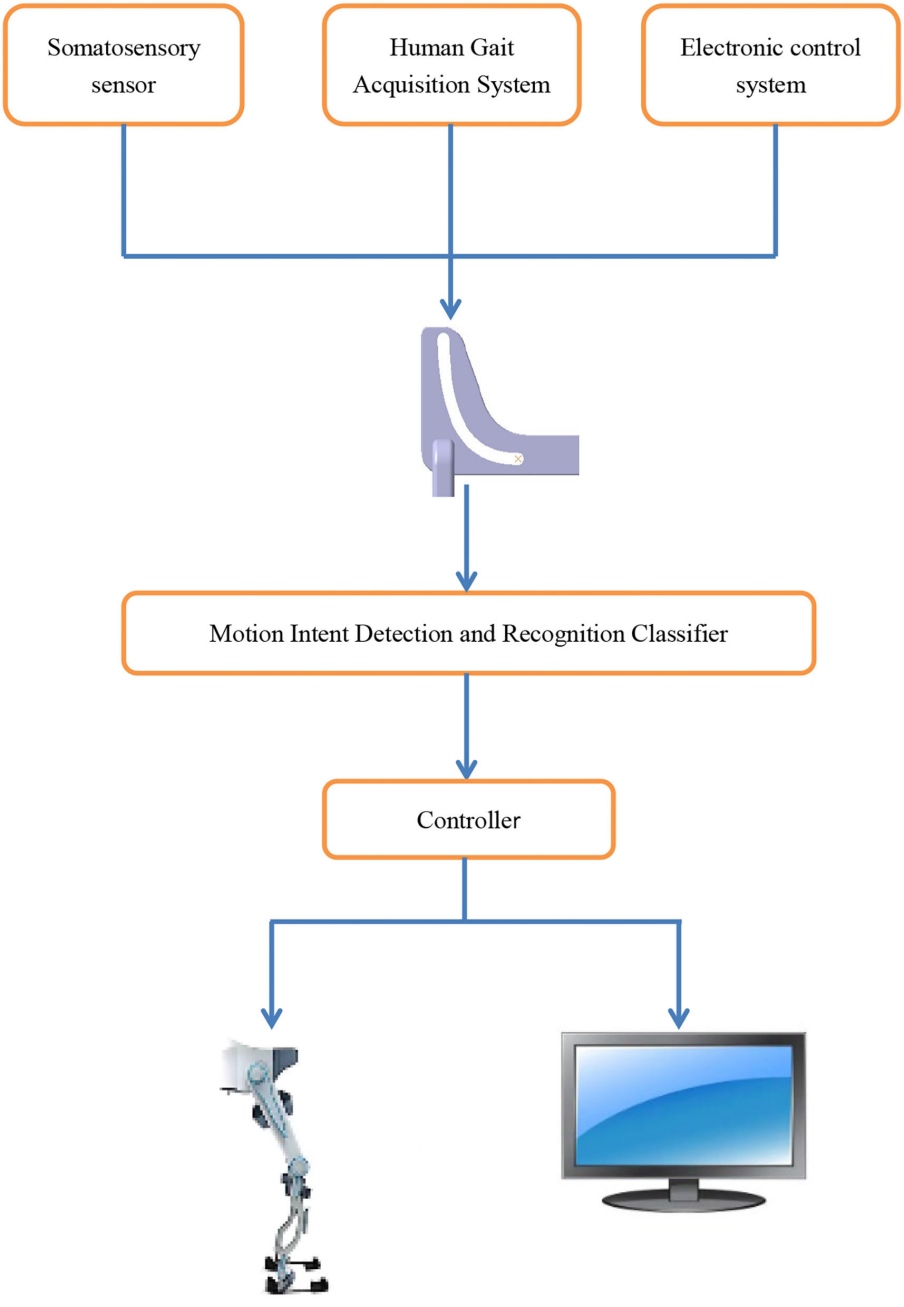
Application in lower limb skeletal robot.

This research aims to study the STS process and balance ability of the human body through somatosensory sensors and the human gait acquisition system and to analyze the change of center of gravity movement trajectory during the STS process of the human body. Our research objectives are mainly in the following three aspects: First, the comfort and safety of the elderly during STS will be improved while maintaining self-confidence and pleasure, realizing the organic combination of emotion and engineering, and the integration of art and science. Second, the medical problem of the elderly having difficulty in STS will be solved by engineering means, realizing the integration of medical and industry. Third, it will provide the basis for future STS-related auxiliary motion technology and auxiliary motion equipment. As shown in Figure 1, based on the mechanical chute, the motion sensor, human gait acquisition system, and electronic control equipment are equipped and then applied to the lower limb skeleton robot. The controller can control the time and speed of the three stages from taking off the chair and getting up to standing so as to achieve the function of stably assisting standing.

## Research Methods

In our research, we determined the trajectory of the center of gravity movement during STS with the Vicon system and analyzed the velocity of the human STS process. Then, the velocity of the STS process was measured by combining the dynamic profile of the AMTI plantar pressure sensor and the center of gravity movement profile during the STS process, relying on the rules of health management and service management. In addition, we analyzed the non-equilibrium motion states that are harmful to the human body.

Vicon motion capture system produced by OML is used to collect data with Vicon optical gait. Vicon MX motion capture camera and other components connected by the network constitute a complete 3D motion capture system to collect real-time optical data. Optical data are formed by pasting feedback at the marking point. The whole-body model of Vicon software Nexus 1.8.5 was used in the experiment, and a total of 39 points were glued to the human body.

The AMTI 3D force measuring table developed by AMTI USA was used for the only pressure test. Several resistivity sensors were installed on the force table to measure the pressure variation in different parts of the entire platform (Li, 2012). After calculation, the force and torque variations on the X-, Y-, and Z-axes can be obtained. A number of two 40 x 60 cm pressure tables were used in this study.

This study was conducted for the needs of the elderly. However, young people were chosen as the subjects because of the risks associated with the forward fall experiment and the difficulty in grasping the degree of undressing and labeling for the Vicon gait capture preparation experiment. The subjects recruited for this study completed the sitting to standing and forward fall experiments under the three test procedures described above requirements, and the relevant data were obtained. The seat's height was 45 cm, and each person stood three times. For fall detection, the pre-fall test was used in this study.

Emotional states such as comfort are reflected by the safe speed and angle during STS.

In this study, the Kinect sensor and the Vicon system were used to measure the trajectory of the center of gravity movement during STS. The Vicon system is used to explore the safety of human STS movement and to stage the STS movement by combining the AMTI planar pressure sensor with the dynamic curve of the center of gravity. The processing speed of STS posture is completed from health management and service management specifications.

**Figure 2:**
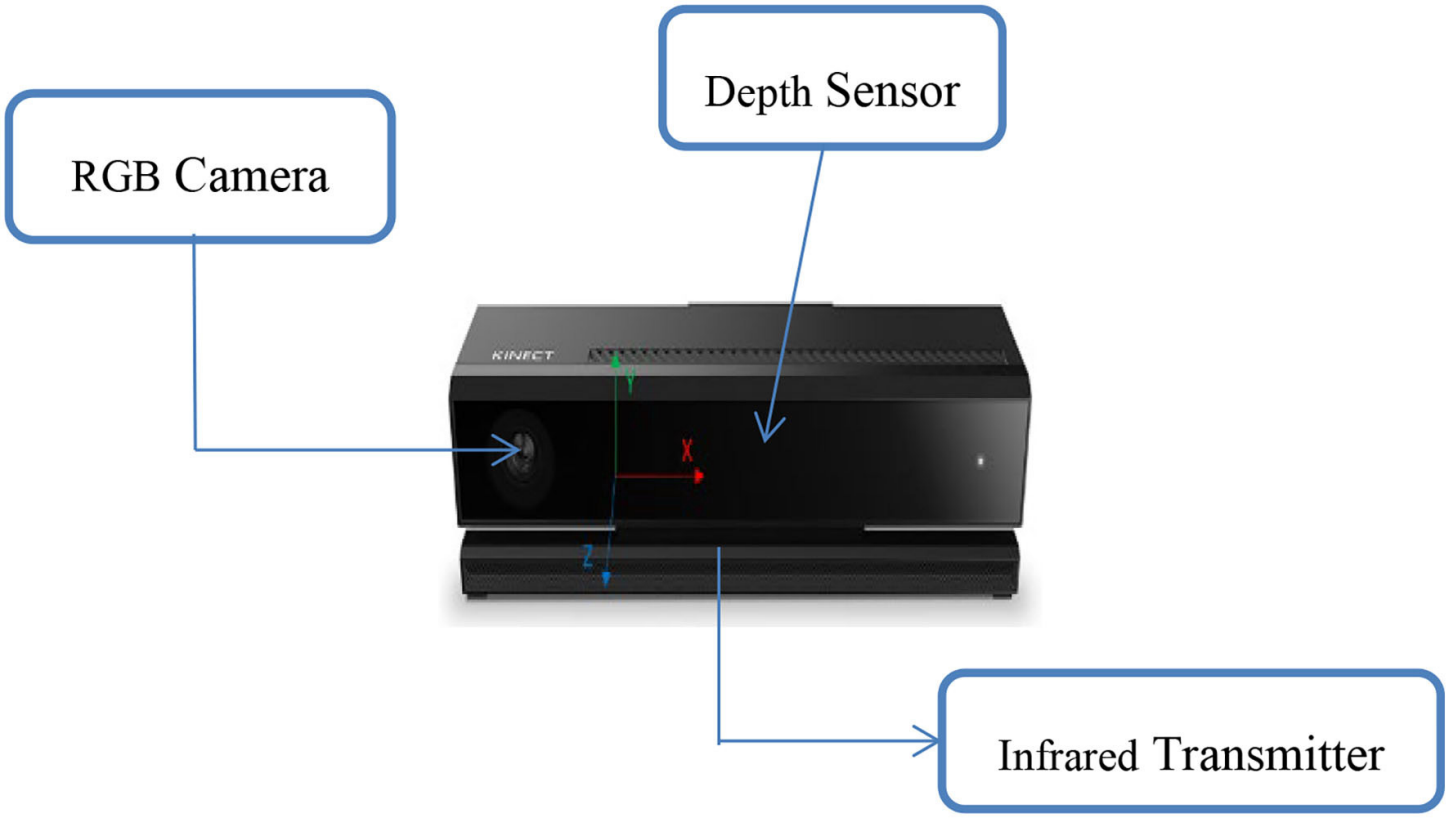
Kinect 3D bone coordinate measuring equipment.

**Figure 3:**
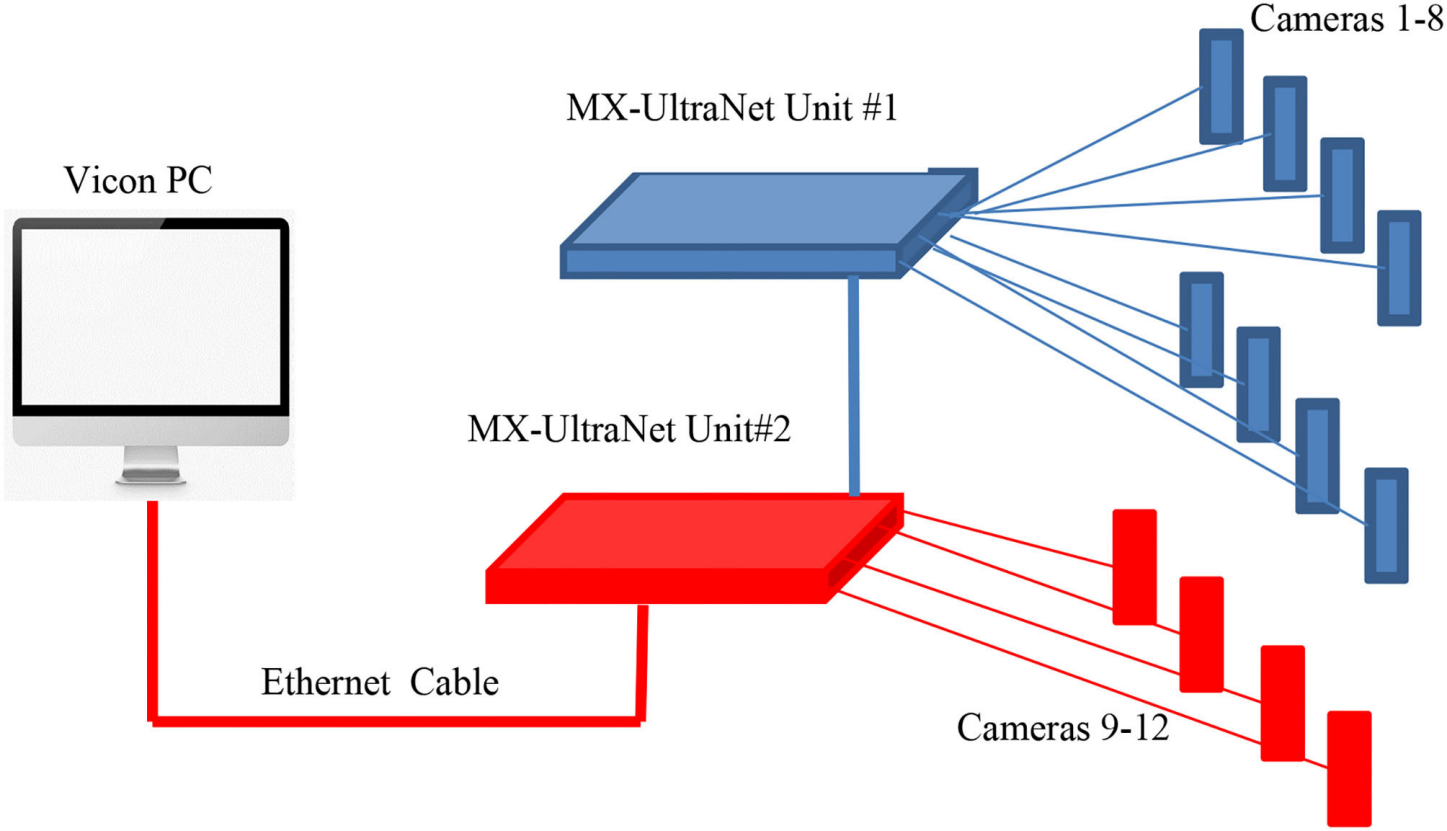
Vicon optical gait acquisition system.

At present, the following methods are mainly used to study the trajectory of the body center of gravity in the world: estimation method, balance plate method, Vicon optical gait acquisition system, and Kinect three-dimensional bone coordinate measurement. However, although the estimation method is simple in principle and convenient in operation, it lacks accuracy, personalization, and precision, so it is not adopted in this study. This study mainly relies on the bone tracking technology of Kinect to measure the center of gravity of human movement during STS. The determination method is to synthesize the three-dimensional coordinate data of each joint in segments and finally converge into the trajectory of the moving center of gravity of the whole body (trajectory). The Vicon optical gait acquisition system is used to measure the trajectory of the center of gravity of the human body in STS motion. We compare it with the trajectory of the Kinect sensor to verify the measurement results and explore the safety. Combined with the plantar pressure test, each phase of the standing motion process was subdivided and analyzed to explore the human body's safety and determine the safe, threshold, and dangerous speed. Figures 2, 3 show the schematic diagram of the Kinect 3D bone coordinate measuring instrument and Vicon optical gait acquisition system, respectively.

**Figure 4:**
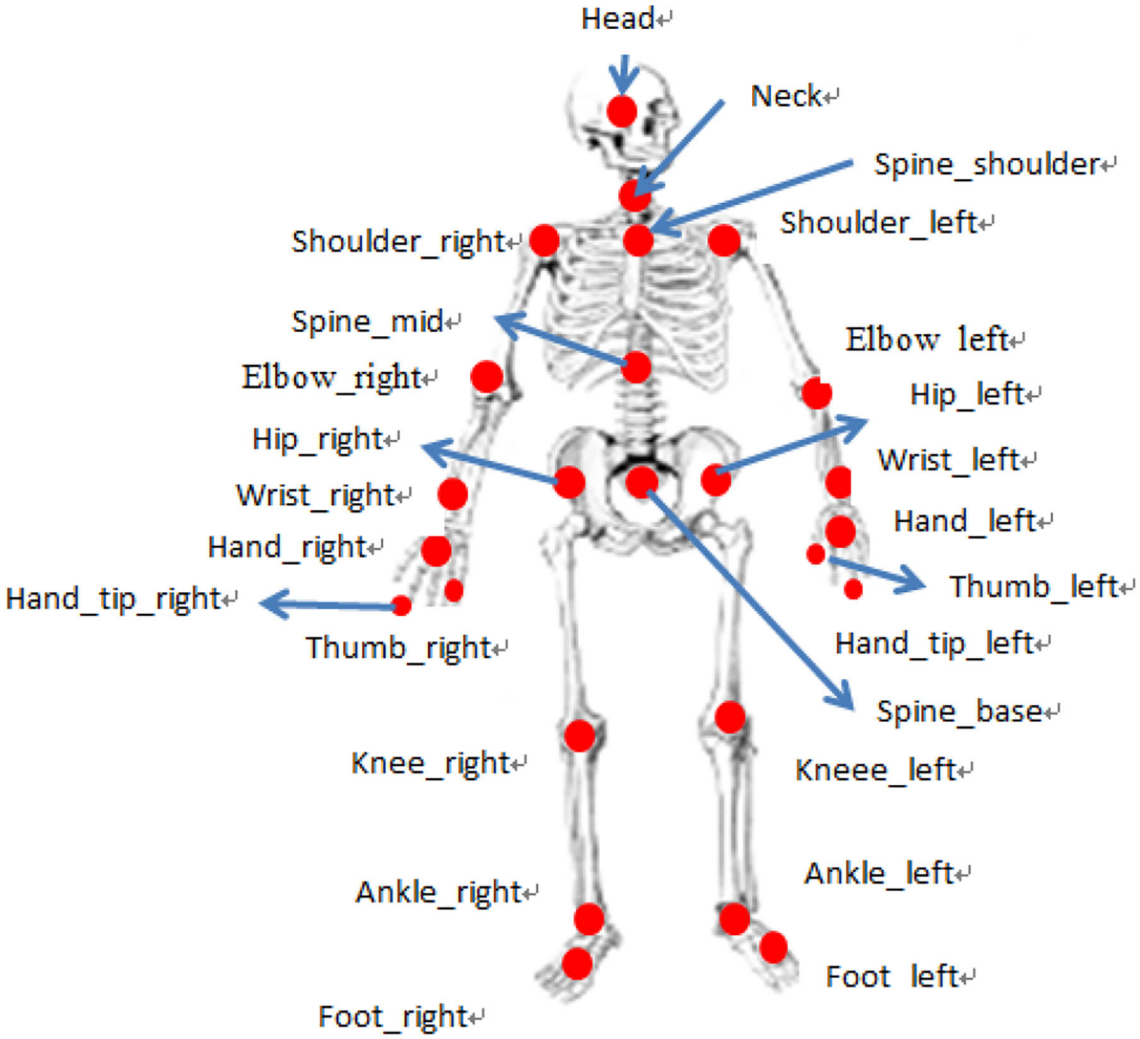
Kinect human skeleton joint model.

When using Kinect skeleton tracking technology, the Skeleton Stream in the Kinect for Windows SDK, a Kinect development application, provides data for 25 key nodes of the human body (Jian, 2013), as shown in Figure 4. To use the skeleton data, we adopted skeleton tracking technology. Once the skeleton tracking program is initialized, we can obtain the desired 3D joint data from the Skeleton Stream. Each set of joint 3D coordinate data in Skeleton Stream is a set of 3D coordinate data information of the skeleton, which includes the bone position and the 3D coordinate data of the joint. Each joint has a specific label, such as knee joint, shoulder joint, frontal joint, wrist joint, and other information and 3D coordinate data (Hof, 2004).

According to Figure 2, the coordinate origin (x = 0, y = 0, z = 0) of the Kinect coordinate system is located in the center of the Kinect infrared camera. The X-axis direction is the left direction of Kinect irradiation direction, the Y-axis direction is the upper direction of Kinect irradiation direction, and the Z-axis direction is the Kinect irradiation direction. The coordinates are in meters (m). The camera space refers to the 3D spatial coordinates used by Kinect.

Based on Kinect skeleton tracking technology, the center of gravity of the STS movement is determined. The determination method is to synthesize the center of gravity motion trajectory of the whole body by piecewise synthesis of the 3D coordinate data of each joint.

**Figure 5:**
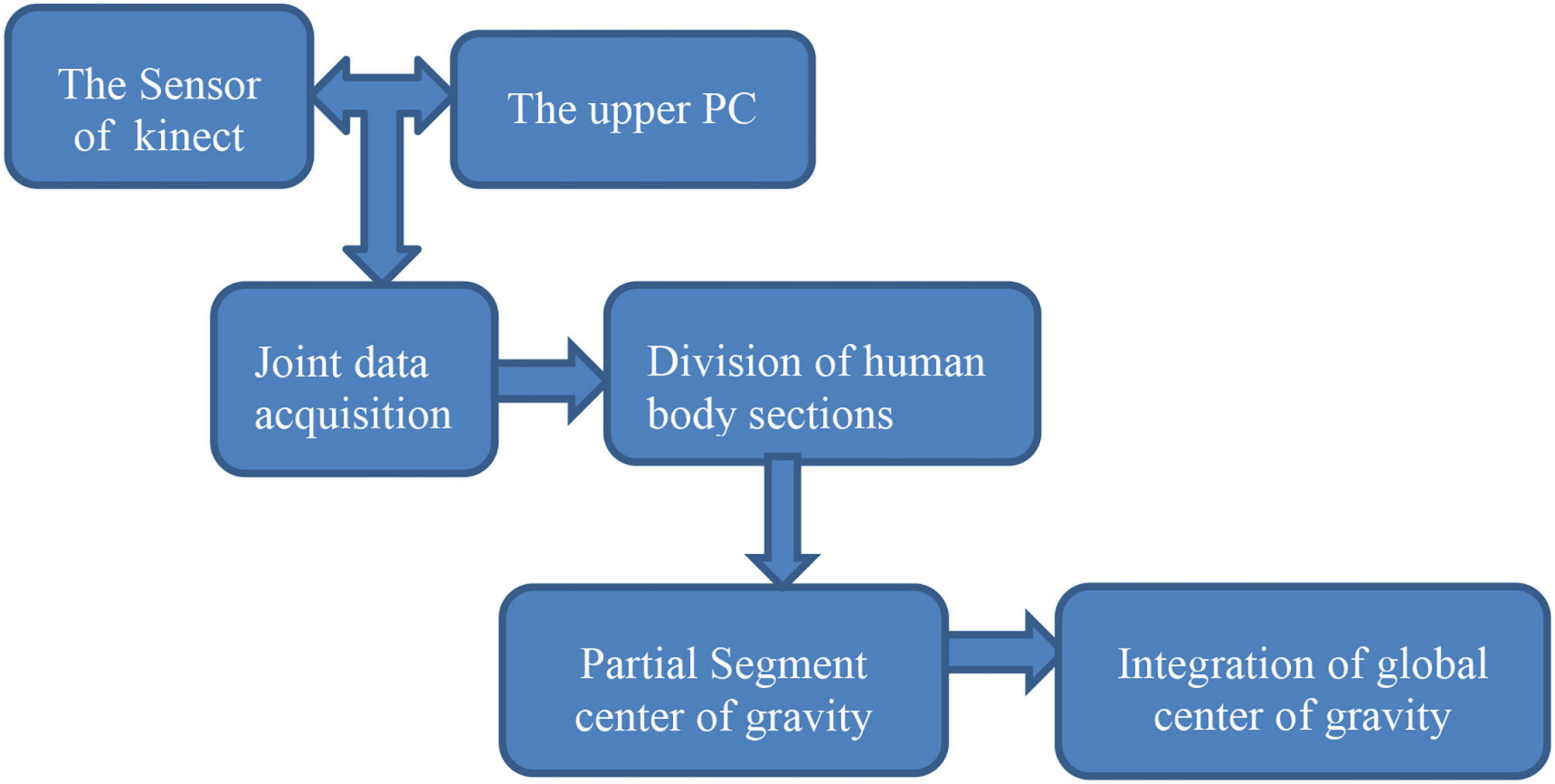
Composite block diagram of center of gravity.

The algorithm flow of center-of-gravity synthesis is in Figure 5.

**Figure 6:**
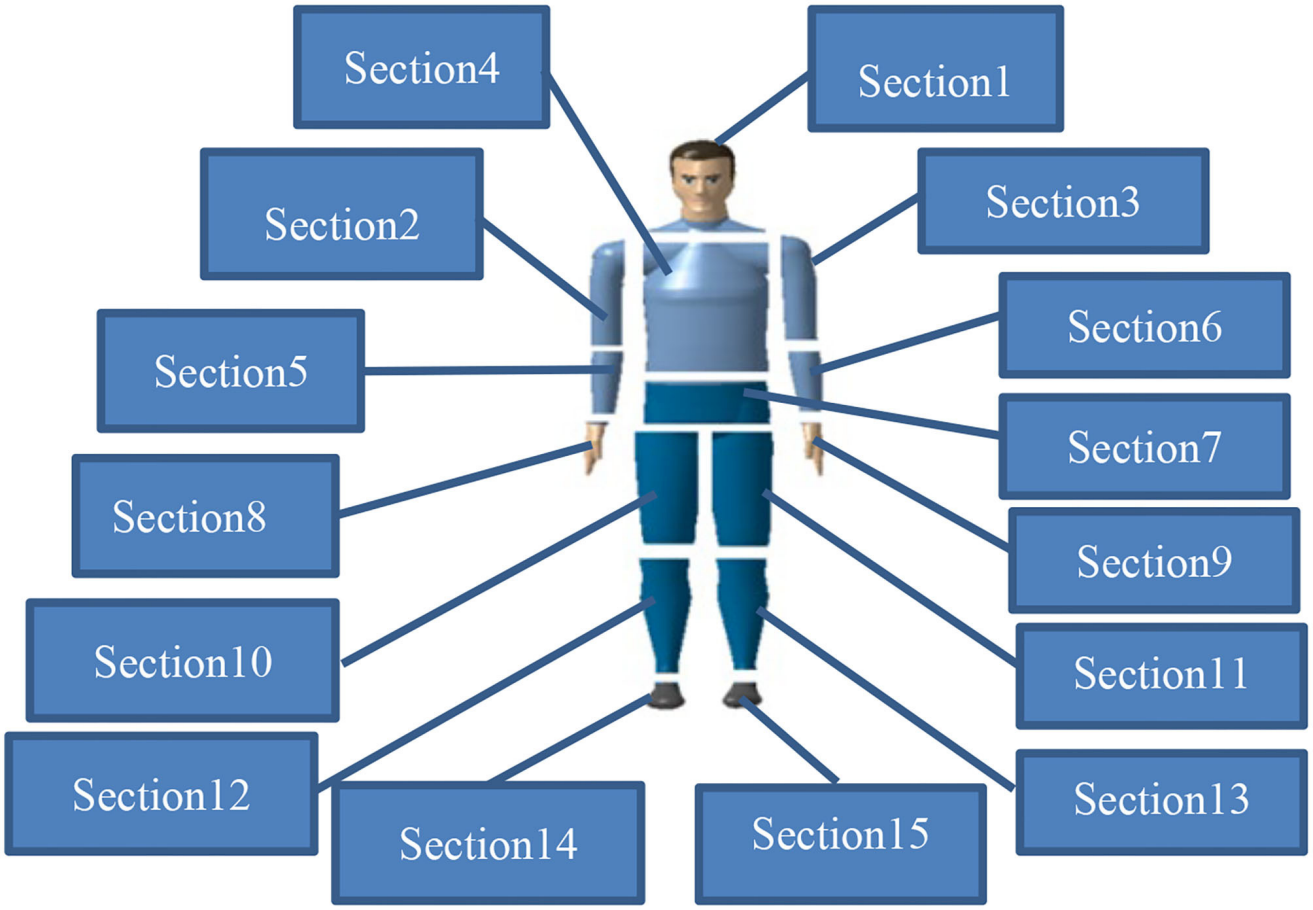
Division of 15 sections of human body.

Based on the human body model and the motion structure of the whole body, this segmentation method first divides the human body into 15 regional segments, as shown in Figure 6. The segmentation results and correlation coefficients are shown in Table 1. Both the Kinect skeletal tracking system and the Vicon motion capture system determine the coordinates of the distal and proximal parts of each regional segment of the human body from the collected data. Then, we define the COM of each regional segment with the following formula:


$$\begin{array}{l} x=x_{p}q_{p}+x_{d}q_{d}\\ y=y_{p}q_{p}+y_{d}q_{d}\\ z=z_{p}q_{p}+z_{d}q_{d} \end{array}$$

In the formula, x, y, and z, respectively, represent the coordinate value of *COM*_*i*_ of each regional segment of the human body in the above coordinate system. The distal end of each region of the body from the center is called the distal end, and the proximal end of each region of the human body from the center is called the proximal end. *x*_*p*_,*y*_*p*_,*z*_*p*_ represent the proximal coordinates, *x*_*d*_,*y*_*d*_,*z*_*d*_ represent the distal coordinates, *q*_*d*_ is the distal coefficient, and *q*_*p*_ is the proximal coefficient.

**Table 1:** Division and correlation coefficients of human body segments.

**The body section**	**Far end, near end**	**Body segment mass coefficient** **k_i_(M_i_/*M*)**	**Far-end coefficient q_d_**	**Proximal coefficient q_p_**
Head and neck	Center of left and right temporal ridge, center of left and right acromion	0.081	0	1.000
Chest and abdomen	Left and right acromion center, left and right iliac crest center	0.355	0.500	0.500
The arm	Left and right shoulders, right and left elbows	0.028	0.564	0.436
Forearm	Left and right elbow and wrist	0.016	0.570	0.430
Hand	Left and right wrists and hands	0.006	0.494	0.506
Pelvis.	Center of pelvis, center of left and right thigh	0.142	0.895	0.105
The thigh	Left and right thighs, left and right knees	0.100	0.567	0.433
The calf	Left and right knees, left and right ankles	0.0465	0.567	0.433
Foot	Left and right ankles, right and left balls of feet	0.0145	0.500	0.500

Synthesis of integral COM is to synthesize the partial center of gravity obtained from the above 15 regions of the human body to obtain the overall center of gravity of the human body, which can be obtained by the following formula (Li, 2012):


$$\begin{array}{l} COM=\frac{M_{i}}{M}\overset{i=n}{\underset{i=1}{\sum }}COM_{i}=k_{i}\overset{i=n}{\underset{i=1}{\sum }}COM_{i} \end{array}$$

Where COM is the total center of gravity of the human body, *COM*_*i*_ is the center of gravity of each section, M is the total mass of the human body, *M*_*i*_ is the mass of each section of the human body, *i* is the number of body segments, and *k*_*i*_ is the specific gravity coefficient of each section of the body.

Kinect's development environment is Windows 10, and the depth camera in Kinect 2.0 captures data at 30 frames per second.

**Figure 7:**
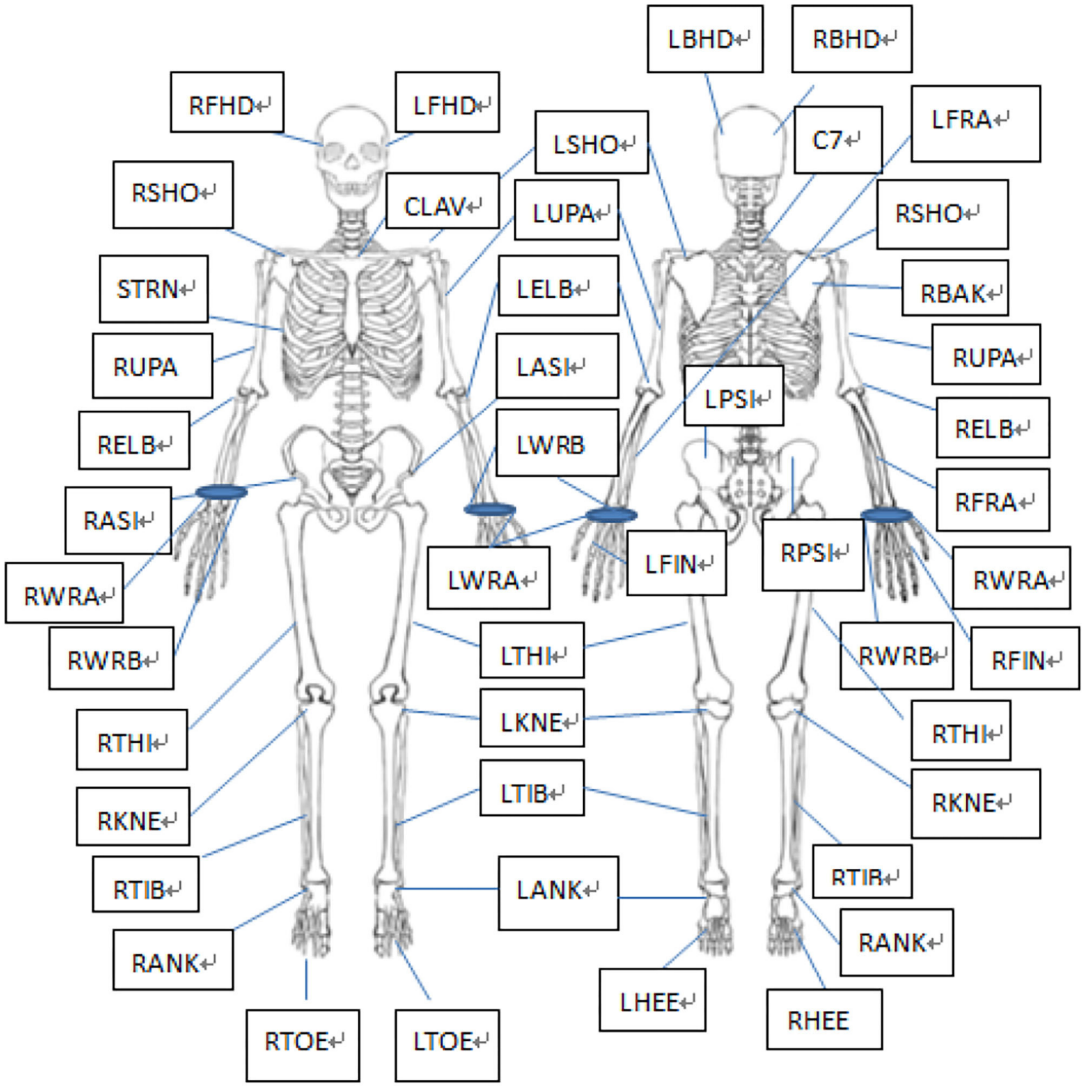
Paste diagram of marking points.

Vicon motion capture system from OML is used to collect Vicon optical gait data. Vicon MX motion capture camera and other components connected by the network constitute a complete 3D motion capture system to collect real-time optical data. Optical data are formed by pasting feedback at the marking point. The sticking points of the human body are shown in Figure 7 (39 points in total): the head contains 4 points: left front, right front, left rear, and right rear; the trunk contains five points: the 7th cervical vertebra, the 10th lumbar vertebra, the upper part of the manubrium sternum, the lower part of manubrium sternum, and the middle part of right scapula. The upper limb contains 14 points, 7 on each side: acromion end, upper arm, elbow joint, forearm, medial wrist joint, lateral wrist joint, and first phalangeal joint; the pelvis contains four points: left and right anterior superior iliac spine and left and right posterior superior iliac spine; the lower extremity contains 12 points, with 6 points on each side: knee joint, thigh, calf, ankle, toe, and heel.

**Figure 8:**
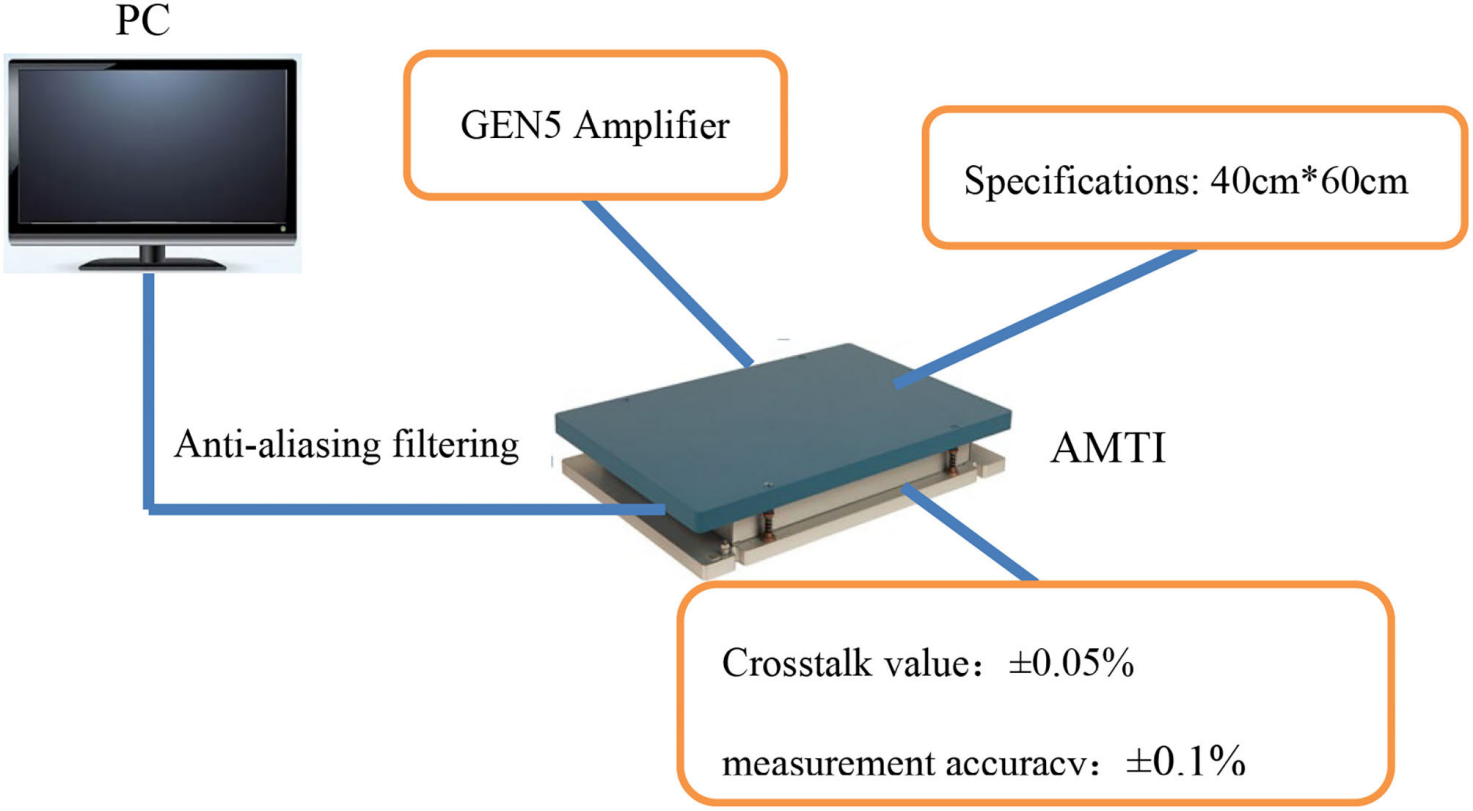
AMTI three-dimensional force table.

The AMTI three-dimensional force measuring table developed by the American AMTI company was used for the sole pressure test. Several piezoresistive sensors were installed on the force table to measure the pressure variation across different parts of the platform (Zhifei, 2011). After calculation, force and torque changes on X-,Y-, and Z-axes can be obtained. The force table uses high sensitivity piezoresistive sensor to measure the tiny pressure changes of the human body in various postures, which can be accurately converted into force, torque, and other values. In this paper, two pressure tables of 40 x 60 cm are used. The equipment is widely used in clinical medicine, sports science, human–computer interaction, aerospace, and other fields. The device is shown in Figure 8.

## Research Results and Discussion

**Figure 9:**
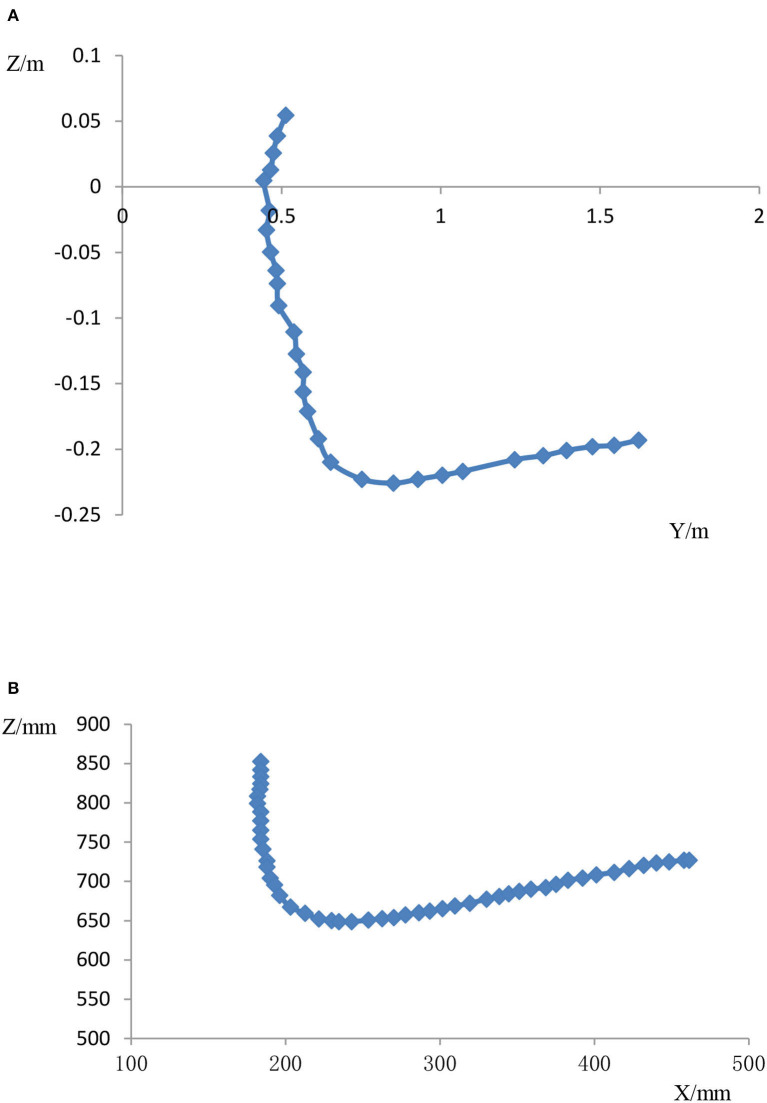
Center of gravity movement trajectory during STS. **(A)** Human COM curves collected by Kinect. **(B)** Human COM curves collected by Vicon.

Through the above research methods, Kinect and Vicon were used to measure the center of gravity movement trajectory of the elderly during STS, as shown in Figure 9.

The left and right figures show the human body's center of gravity movement curves collected by the Kinect and Vicon systems, respectively. We compared the center of gravity change curves of the two human bodies during STS and calculated them by the algorithm. It was found that the two-axis correlations of the human center of gravity trajectories for the Kinect and Vicon systems were 0.982658 and 0.984427, respectively. The dynamic human trajectory tracked by the Kinect system is highly correlated with the trajectories of the high-frequency, high-precision measurement data from the Vicon system, and the trend is consistent. This result tells us that for the same STS process, both the bone point coordinates captured by the Kinect system and the human body's three-dimensional COM collected by the Vicon system can well reflect the COM motion trajectory in STS motion. Considering the occlusion traces of the Vicon system during the STS process, the Kinect system can better compensate for this deficiency, whereas the high frequency and high accuracy of the Vicon system compensate for the low measurement accuracy of the Kinect system. By combining the two, the parameters related to the complete STS motion can be obtained more effectively. When these two methods are applied to the measurement of the same sit-to-stand process, the dynamic COM position of the human body can be reflected more accurately. Meanwhile, in this study, compared with other methods, our motion trajectory measurement method has higher accuracy, large and complete data volume, and a simple experimental measurement process. So, we obtain a more realistic reflection of the dynamic COM motion trajectory of the human body during STS. In addition, we compared the measured dynamic COM curves with the hip motion trajectories of normal subjects doing STS movements provided by the University of Ljubljana, Slovenia (Hughes and Schenkman, 1996) and found some strong correlations. It proves the reliability and authenticity of the obtained COM curves in another way. Through the research and experiments, we obtained the center of gravity motion curves and related dynamic parameters that can truly reflect the STS situation of the elderly. This provides a basis for the subsequent research of related auxiliary sit-to-stand devices, such as auxiliary chairs, auxiliary toilets, and auxiliary standing arms, and the development of auxiliary technologies.

**Figure 10:**
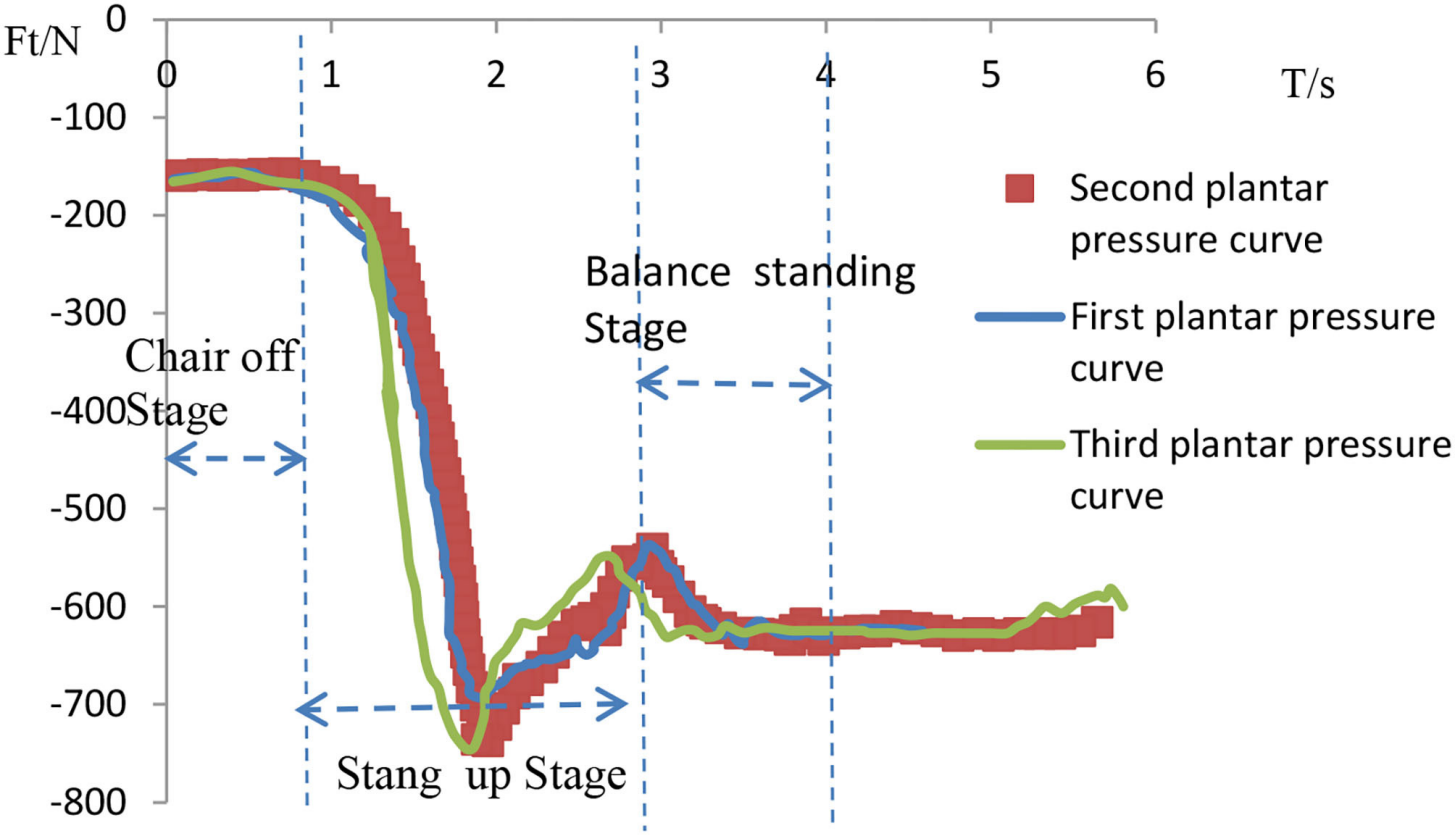
Subjects standing under a 45-cm seat with plantar pressure curve.

**Figure 11:**
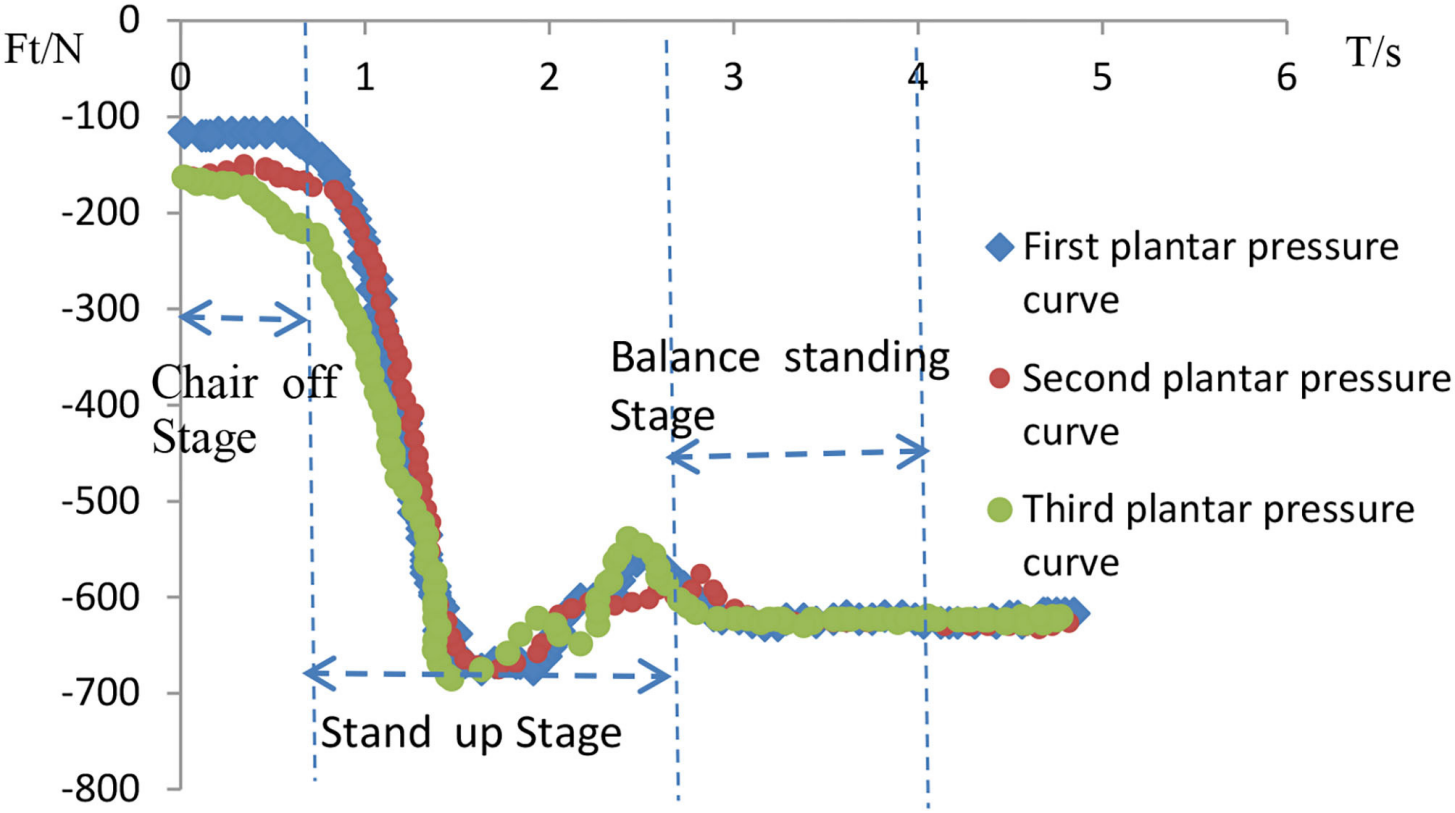
Plantar pressure curve of subjects standing under the seat at 55 cm height.

**Figure 12:**
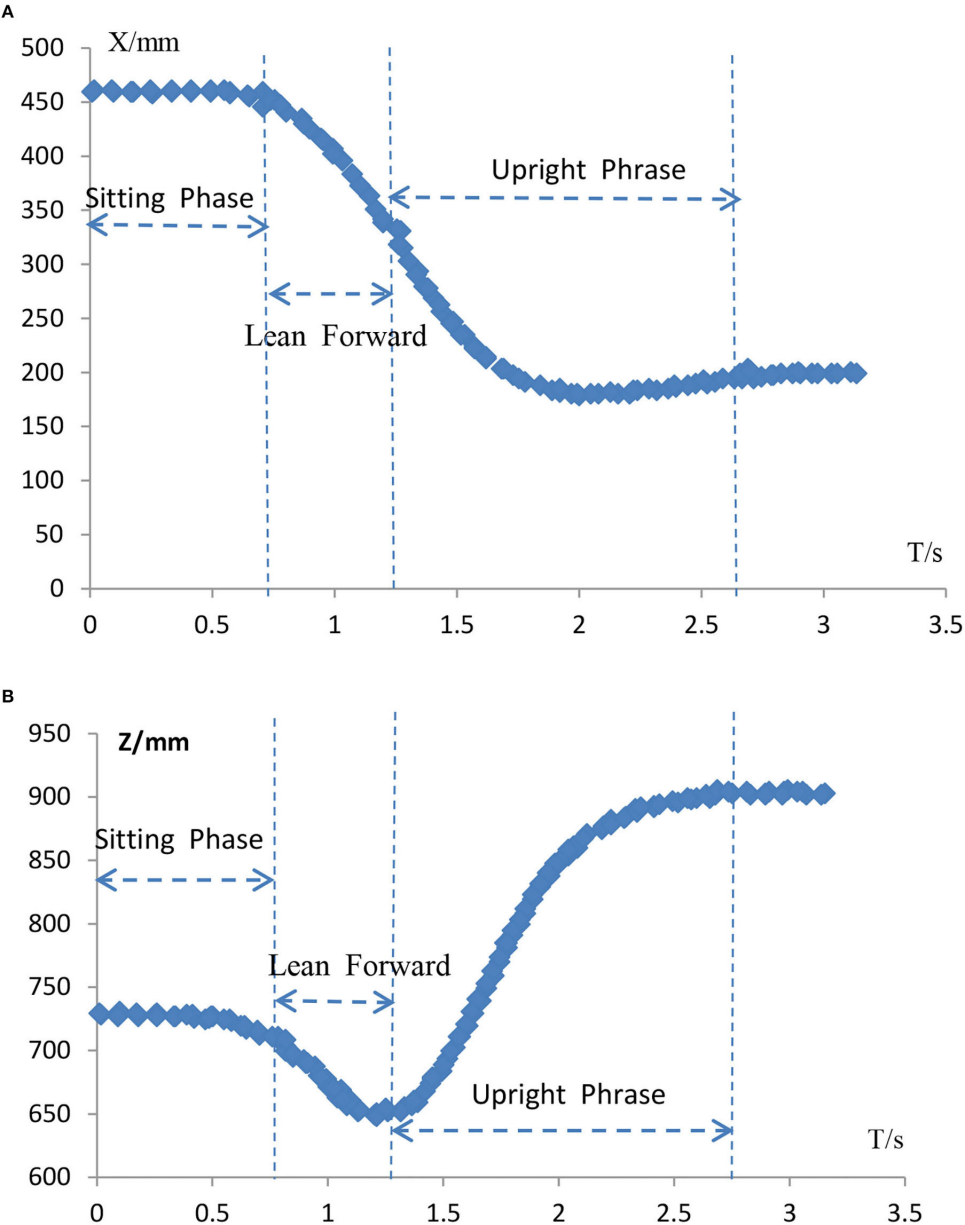
Vicon dynamic COM curve of human body. **(A)** Change in X-axis displacement. **(B)** Z-axis displacement change.

**Figure 13:**
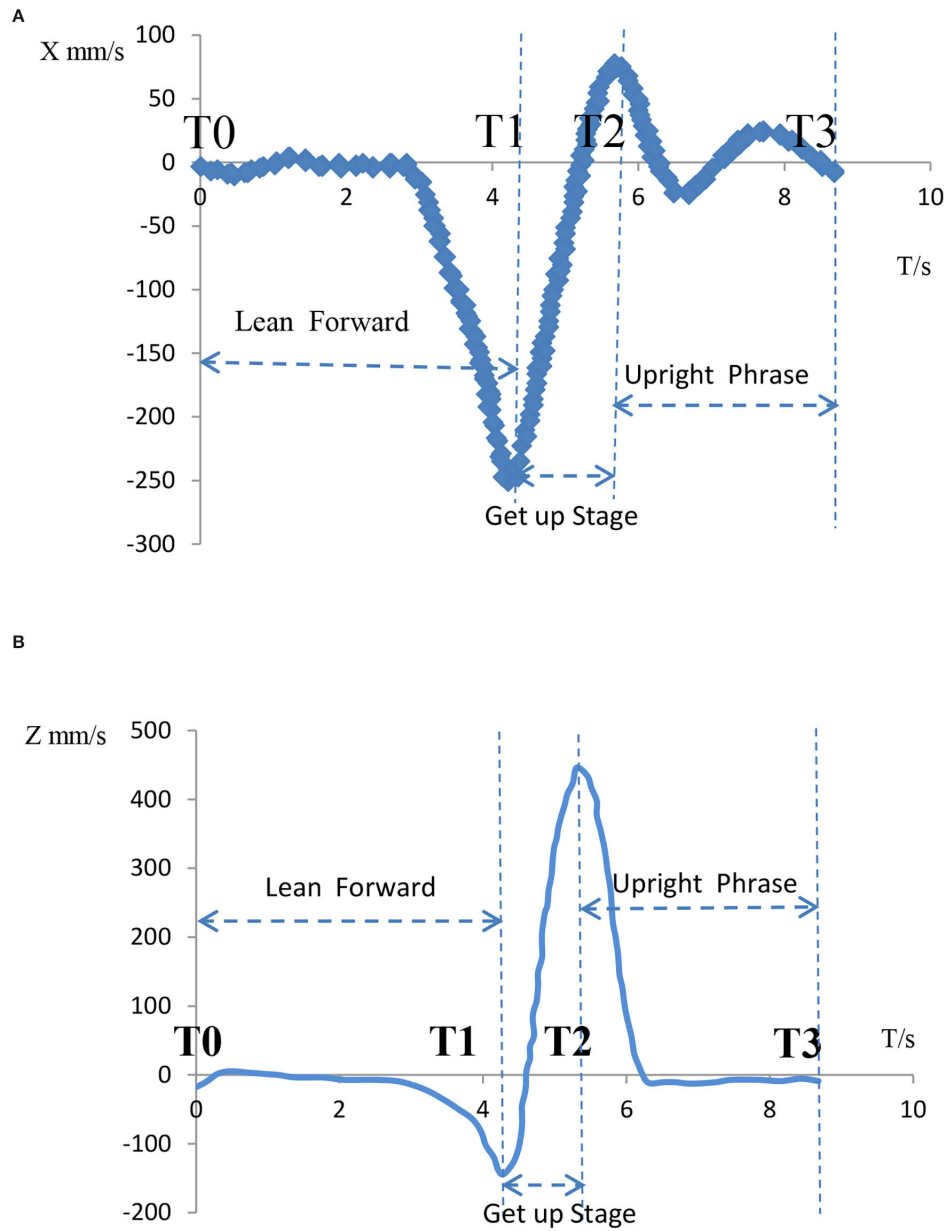
Velocity change of X/Z axis of human body's center of gravity. **(A)** Change in X-axis velocity. **(B)** Change in Z-axis velocity.

Figures 10–**13** show the state data obtained from the Vicon system and the AMTI plantar pressure sensor. As can be seen in Figures 10, 11, the curves show the variation process of the three average plantar pressures obtained by the subjects' sit-to-stand three times on a chair at 45 cm and 55 cm height, respectively. Based on the changes in foot pressure during the STS exercise, we can analyze the pressure changes and the time limit of the STS process during the whole STS exercise and set the optimal reference value for the initial height of the STS process.

As shown in curves in Figures 10, 11, the time required for the subjects to complete the STS exercise normally in the seat with a height of 45 cm and 55 cm was about 2.5 and 2 s. Depending on the uncontrollable speed of the subject's STS movement, the time to complete the sit-to-stand fluctuated within 2.5 s. As shown in Figures 10, 11, the plantar pressure increased rapidly during the STS movement when the buttocks left the seat, essentially completing the movement out of the seat at about 0.8 s. This process is critical and requires the most effort. In addition to overcome their own weight, the subjects also need to maintain the strength to continue the standing process, reaching the maximum value of 730 N on the curve. This is because the pressure plate exerts a reaction force on the COM movement of the human body and gives a large momentum in the vertical direction, which includes the subjects' own weight and continuous standing power. After that, subjects overcame the effect of their own gravity to complete the STS movement and kept their balance at a constant safe speed within 1 to 1.8 s. Subjects of different body weights should increase or decrease force in proportion, but the safe speed of completion remains unchanged.

The movement from sitting to standing is a continuous and complex movement whose phases are subdivided into various forms. It can be analyzed from a single human center of gravity displacement curve and divided in the form shown in Figure 12. Figure 12A shows the displacement curve along the X-axis, and Figure 12B shows the displacement curve along the Z-axis. Each COM curve can be roughly analyzed in three sections. The first section is the sitting phase before standing up in preparation for standing up. In the second stage, the body leans forward to prepare for leaving the chair. At this point, the center of gravity in the Z-axis gradually drops to its lowest level. This stage lasts for a short time to reserve large kinetic energy to stand up. The third phase is the standing phase, in which the knee joint has to overcome a large torque, requiring a high level of leg strength, and this phase lasts about 1.5 s. Therefore, the third stage is the necessary stage of exertion and the critical stage for standing up.

From the perspective of the velocity of the center of gravity, we divided the whole process of STS motion into the following aspects. Figure 13 shows the velocity curves of the X-axis and Z-axis of the human center of gravity during the STS process. According to the time nodes, the STS motion can be subdivided into the following three periods, T0–T1, T1–T2, and T2–T3, which are the three motion states of leaning forward, standing (the buttocks completely leaving the seat), and resuming uprightness, respectively. Looking at the velocity changes during the study of getting up and leaving the seat and resuming upright posture, the curves in Figure 13 show that the velocity of each axis increased rapidly during T1–T2. During this phase, the leg muscles need to overcome the effect of human gravity, and the whole phase of getting up and leaving the seat is basically completed around 0.8–1 s. The velocity of the X-axis decreases from 0 to −230 mm/s during the T0–T1 phase and then increases to 70 mm/s during the T1–T2 phase. The velocity of resuming the upright state fluctuates around 0 mm/s during the T2–T3 phase. The velocity of the Z-axis decreases from 0 mm/s to −150 mm/s during the T0–T1 stage, then dramatically increases to 450 mm/s in the T1–T2 stage, and rapidly decreases to 0 mm/s in the T2–T3 stage.

According to the X/Z axis data, the safe speed of the horizontal axis during standing can be reduced to 72.8 mm/s when reaching the third stage, and the safe speed can be set as the safe threshold speed. However, it is worth noting that the slow standing speed will hurt the knee joint and muscles of the elderly, so that the Z-axis speed can remain unchanged.

**Figure 14:**
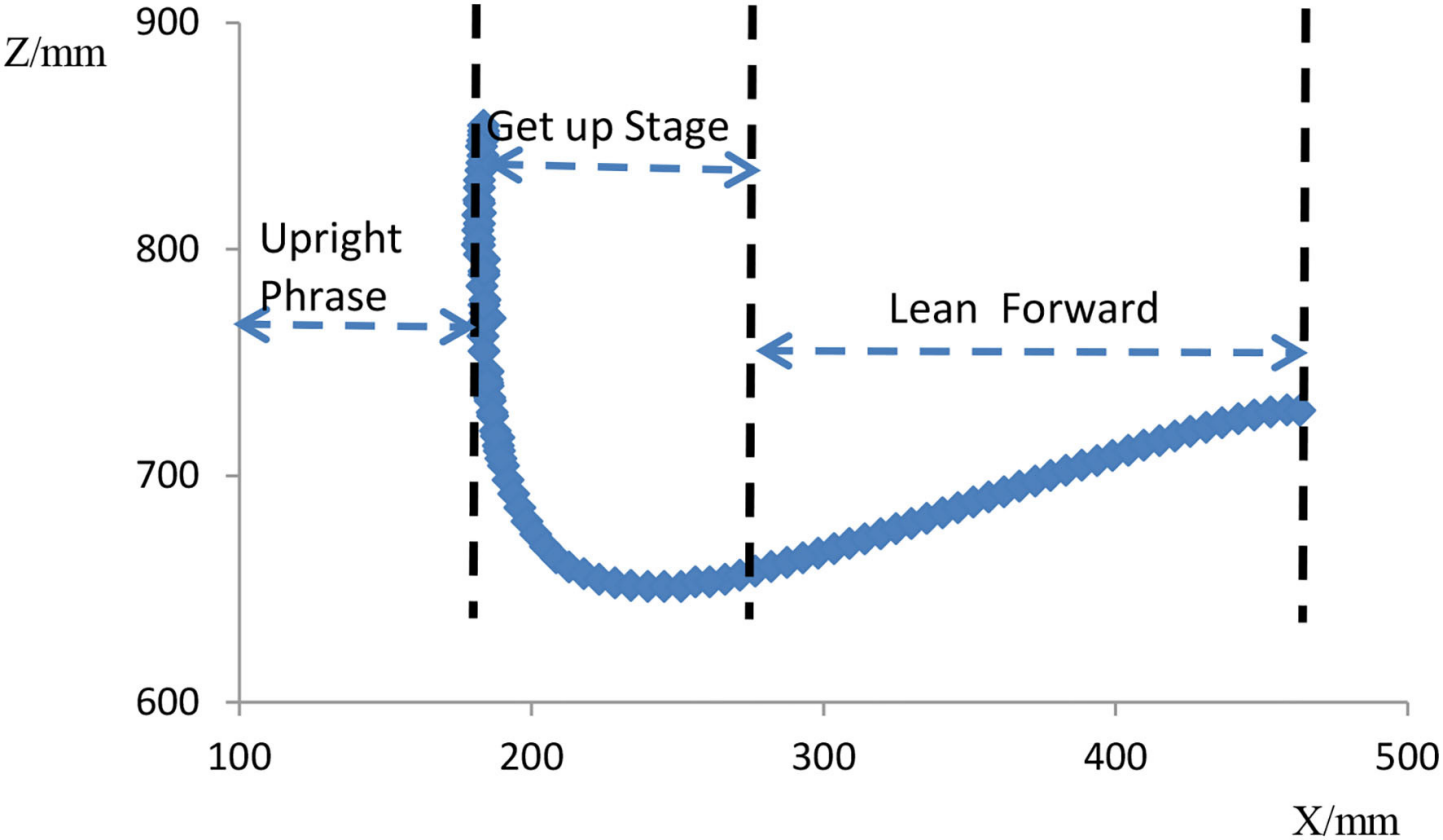
Center of gravity movement trajectory and stage division during standing process.

**Figure 15:**
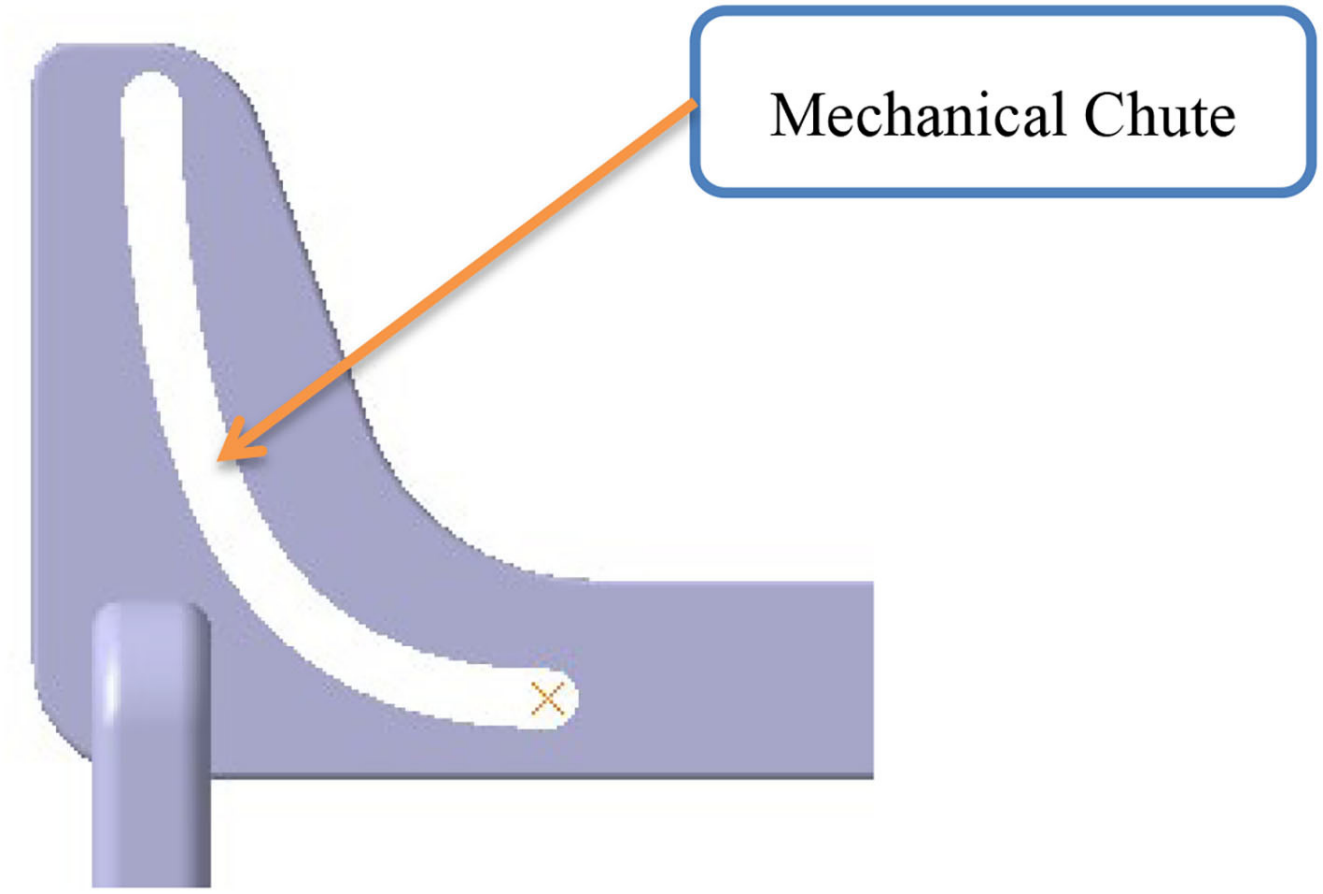
Schematic diagram of mechanical chute mechanism.

In addition, the height of the standing posture was 45 to 55 cm at the beginning of the standing process. During the STS movement of the subjects, we measured the dynamic trajectory of the human COM and found that the COM had a large slope during the ascent phase. The lower the height of the seat, the greater the slope. The greater the slope, the easier it is to stand up and the less psychological impact on the elderly. As we found in the literature, Tianyi Wang et al. from Osaka University, Japan, studied the effects of assisted rehabilitation robots on people and proposed that autonomous STS movements should be performed with 43 and 62 cm height seats. The results showed that completing the task was comfortable and easy under a 62-cm seat while standing up was difficult under a 43-cm seat. Furthermore, Huges et al. from the University of Kansas Medical Center suggested that the difficulty in STS exercises decreases with increasing seat height within the adjustable range of seat height. In addition, they also found that older adults have difficulty in completing STS exercises at the height of 38 cm. This result is in full agreement with our findings. However, it is important to note that too high a seat height for completing the posture can be uncomfortable. Therefore, we set the initial height of the seating position at 45 to 55 cm and not more than 55 cm. Movement is the ultimate requirement for health management and services. We combined the initial height, speed, and time control of the standing process with the COM movement curve of the human body during the STS movement obtained above to make the standing process both perfectly completed and safe, providing excellent comfort for the elderly. For example, based on the trajectory curve of gravity transfer during STS measured by the Vicon system, we further subdivide the STS process into three phases (Figure 14), namely, the forward-leaning phase, the standing up phase, and the return to uprightness phase. It can evolve into a mechanical slide structure (Figure 15). The track of the sliding process of the mechanical chute is the track of the STS process for the elderly. Based on this, electric control equipment is configured to control the completion time and speed of the three stages to achieve a comfortable and safe standing environment for the elderly.

## Conclusions

Through research, we obtain a movement curve and related dynamic parameters that can genuinely reflect the STS situation of the elderly. Through the analysis of the speed limit of the STS of the elderly, the speed limit is the key to overcome the psychological barrier of the STS for the elderly, so that the elderly can stand happily, comfortably, and safely. Meanwhile, from the perspective of health management and services, the seat height of the elderly needs to meet the comfort level of the elderly sit-to-stand. Our research summarizes the human COM curve, standing speed, time, and posture height and lays the foundation for the future design of relevant auxiliary standing devices and technologies. Most researchers focus on realizing functions while neglecting the fundamental theories, such as motion trajectory, safety, and comfort. The study also effectively uses the results of Neo-Confucianism to explore the relationship between the results of Neo-Confucianism and people's psychological state, thus realizing the integration of literature and scientific research. By solving the medical difficulty in STS through engineering means, the integration of medical and industrial research is realized. With the development of robot technology, man–machine interaction has been applied more and more. Luo et al. (2020) proposed a hybrid shared control method for a mobile robot with omnidirectional wheels, which A human partner utilizes a six degrees of freedom haptic device and electromyography (EMG) signals sensor to control the mobile robot. Additionally, muscle computer interface (muCI), one of the widespread human–computer interfaces, has been widely adopted for the identification of hand gestures using the electrical activity of muscles (Zhou et al., 2020a). Zhou et al. (2020b) proposed a novel horse inspired all terrain eight-wheeled vehicle with four swing arms for transportation in the mountain battlefield. In the future, we can use robot technology to carry out deeper integration research on the health of the elderly. In addition, we will establish a more comfortable and safer way of STS for the elderly.

## Data Availability Statement

The original contributions presented in the study are included in the article/supplementary material, further inquiries can be directed to the corresponding author/s.

## Author Contributions

All authors listed have made a substantial, direct, and intellectual contribution to the work and approved it for publication.

## Funding

This project was supported by the Open Project of China International Science and Technology Cooperation Base on Intelligent Equipment Manufacturing in Special Service Environment, Project No.: ISTC2021KF07. This research was also funded by Provincial Teaching Research Project (Health Service and Management Curriculum System Construction, 2019JYXM0879) and 2020 Outstanding Top-Notch Talents Training Fund for Universities (gxbjZD2020034).

## Conflict of Interest

The authors declare that the research was conducted in the absence of any commercial or financial relationships that could be construed as a potential conflict of interest.

## Publisher's Note

All claims expressed in this article are solely those of the authors and do not necessarily represent those of their affiliated organizations, or those of the publisher, the editors and the reviewers. Any product that may be evaluated in this article, or claim that may be made by its manufacturer, is not guaranteed or endorsed by the publisher.

## References

[B1] Bai J. J. (2010). Design and Research of Multi-Functional Nursing Service Robot. Nanchang University. Available online at: https://kns.cnki.net/KCMS/detail/detail.aspx?dbname=CMFD2011&filename=2010247633.nh

[B2] China Pension Network. (2018). Analysis of the Current Situation of China's Aging Population in 2018 Problems Brought About by Aging and Countermeasures. Available online at: WWW.CNSF99.COM (accessed August 04, 2018).

[B3] Dall P. M. Kerr A. (2010). Frequency of the sit to stand task: an observational study of free-living adults. *Appl*. Ergonom. 41, 58–61. 10.1016/j.apergo.2009.04.005 19450792

[B4] Heqing Q. (2011). Augmented Reality Education Auxiliary System Based on Kinect and Gesture Recognition. Shanghai Jiao Tong University.

[B5] Hernandez V. (2015). Human upper-limb force capacities evaluation with robotic models for ergonomic applications: effect of elbow flexion. Comput. Methods Biomech. Biomed. Engin. 1–10. 10.1080/10255842.2015.1034117 26214374

[B6] Hof A. L. (2004). Comparison of three methods to estimate the center of mass during balance assessment. J. Biomechanics. 37, 1421–1426. 10.1016/S0021-9290(03)00251-3 15275850

[B7] Hughes M. A. Schenkman M. L. (1996). Chair rise strategy in the functionally impaired elderly. J. Rehabilitation Res. Dev. 33, 409–412. 8895136

[B8] Jian H. (2013). Research and Design of Rehabilitation Training Platform Based on Kinect System Motion Acquisition. University of Electronic Science and Technology of China.

[B9] Kamnik R. Bajd T. Williamson J. (2005). Rehabilitation robot cell for multimodal standing-up motion augmentation. IEEE Int. Conf. Robot. 2005, 2277–2282. 10.1109/ROBOT.2005.1570452

[B10] King W. (2010). Available online at: http://www.wisdomking.com/product3230 (accessed March 14, 2010).

[B11] Li G. Y. (2012). Research on Lower Limb Motion Pattern Recognition and Knee Control Method of Dynamic prosthesis. Hebei University of Technology.

[B12] Lihong Z. (2016). Kinematic Synthesis Theory Based on Kinematic Mapping and Its Application in Assisted Rehabilitation Institutions. Hefei University of Technology.

[B13] Lo A. C. Guarino P. D. Richard L. G. Haselkorn J. K. Wittenberg G. F. Federman D. G.. (2010). Robot-assisted therapy for long-term up-per-limb impairment after stroke. N. England J. Med. 362, 177–1783. 10.1056/NEJMoa0911341 20400552 PMC5592692

[B14] Luo J. Lin Z. Li Y. Yang C. (2020). A teleoperation framework for mobile robots based on shared control. IEEE Robotics Automation Lett. 5, 377–384. 10.1109/LRA.2019.2959442

[B15] Min T. Shuo W. (2013). Research progress on robotics. Acta. Automatica. Sinica. 39, 963–72. 10.3724/SP.J.1004.2013.00963

[B16] Qiu-hui W. Yu-kun W. Li-meng L. (2018). Review of rehabilitation robot on research and application. Packag. Eng. 39:83–89. 10.19554/j.cnki.1001-3563.2018.18.018

[B17] Rafii T. H. (2014). Current and emerging robot-assisted en-dovascular catheterization technologies: a review. J. Biomed. Eng. Soc. 42, 697–715. 10.1007/s10439-013-0946-8 24281653

[B18] Wang T. Jeong H. Watanabe M. (2018). Fault classification with discriminant analysis during sit-to-stand movement assisted by a nursing care robot. Mech. Syst. Signal Process. 11, 25–28. 10.1016/j.ymssp.2017.01.051

[B19] Xiaoyu Z. Kaixuan W. (2010). Robot assistive technology, rehabilitation robots and intelligent AIDS. Wisdom King.

[B20] Yuxuan Z. (2016). Design and Experimental Study of Upper Limb Motor Function Reconstruction System Based on Communication Principle and EMG Control. Nanjing: Southeast University.

[B21] Zhifei M. (2011). Simulation and Experimental Research on Control System of Rehabilitation Robot Assisted Standing up. Harbin Institute of Technology.

[B22] Zhou X. He Q. He C. (2020b). Motion kinematics analysis of a horse inspired terrain-adaptive unmanned vehicle with four hydraulic swing arms. IEEE Access. 8, 194351–194362. 10.1109/ACCESS.2020.3033148

[B23] Zhou X. Qi W. Ovur S. E. Zhang L. Hu Y. Su H.. (2020a). A novel muscle-computer interface for hand gesture recognition using depth vision. J. Ambient. Intell. Humaniz. Comput. 11, 5569–5580. 10.1007/s12652-020-01913-3

[B24] Zhou Y. (2016). System Design and Experimental Study of Upper Extremity Motor Function Rebuilding System Based on Communication Principle and EMG Control. Southeast University.

[B25] Zhu L. H. (2016). Kinematic-Mapping-Based Planar Motion Synthesis Theory and Its Application in Rehabilitation Mechanisms. Hefei University of Technology. p.13. Available online at: https://kns.cnki.net/kcms/detail/detail.aspx?FileName=1016270330.nh&DbName=CDFD2017

